# Ophthalmic manifestations at high altitude: A comprehensive study of anterior segment conditions

**DOI:** 10.22336/rjo.2026.08

**Published:** 2026

**Authors:** Anchal Tripathi, Mohini Agarwal, Atul Bhirud, Avinash Mishra, Vinod Baranwal, Debashish Mukherjee

**Affiliations:** 1 Department of Ophthalmology, Military Hospital, Jammu, Jammu and Kashmir, India; 2 Department of Ophthalmology, Military Hospital, Jalandhar, Punjab, India; 3 Department of Ophthalmology, Sitapur Eye Hospital, Sitapur, Uttar Pradesh, India; 4 Department of Oncosurgery, Army Hospital Research and Referral, New Delhi, Delhi, India

**Keywords:** high altitude ophthalmic manifestations, high altitude complications, anterior segment involvement, altitude, duration of exposure, HA = High Altitude, pO_2_ = Partial Pressure of Oxygen, UV = Ultraviolet, AAU = Acute Anterior Uveitis, HSV = Herpes Simplex Virus, HZO = Herpes Zoster Ophthalmicus, CN = Cranial Nerve, CSCR = Central Serous Chorioretinopathy, HAR = High-Altitude Retinopathy, RD = Retinal Detachment, FAF = Fundus Autofluorescence, OCT = Optical Coherence Tomography, BCVA = Best-Corrected Visual Acuity, IOP = Intraocular Pressure, SD = Standard Deviation

## Abstract

**Purpose:**

This study aims to comprehensively analyze ophthalmic manifestations in individuals from low-altitude (< 2,400 meters above sea level) areas who are exposed to high-altitude (HA) environments (3,500 meters above sea level), with a focus on anterior segment conditions and cranial nerve pathologies.

**Methods:**

We conducted a prospective observational study of individuals at HA locations who presented with ocular complaints. Outcome parameters included age, gender, duration of exposure to HA, altitude of the location, duration of symptomatology, best corrected visual acuity (BCVA) at presentation, final BCVA post resolution, diagnosis based on signs and symptoms, treatment undertaken, time elapsed for resolution/hospital stay, associated laboratory findings, recurrences (if any), and need for de-induction/descent.

**Results:**

Of the 2,043 patients who visited the eye outpatient department with ocular complaints, we included 130 lowlanders, with a mean age of 25.67 ± 5.14 years. The mean altitude was 3,560 meters above sea level. The mean duration of stay at HA ranged from 10 weeks to 21 weeks. The anterior segment manifestations that were observed included acute anterior uveitis (AAU) (n=12), Herpes Simplex Virus (HSV) Keratitis (n=30), HSV Keratitis with Iridocyclitis (n=4), Photokeratitis (n=30), Herpes Zoster Ophthalmicus (HZO) (n=10), facial nerve palsy with or without exposure keratopathy (n=24), non-resolving/chronic inflammatory conjunctivitis (n=20). Most cases responded well to treatment. However, 50% of AAU cases, 25% of HSV keratitis cases, 20% of HZO cases, and 33.33% of facial nerve palsy cases recurred within one year and therefore had to be de-inducted to a lower sea level.

**Discussion:**

Delayed resolution was attributed to hypoxia-induced immune dysregulation, increased ultraviolet exposure, and frequent misdiagnosis of red eye at peripheral healthcare settings. High altitude appeared to exacerbate inflammatory and infectious ocular conditions and to precipitate sight-threatening posterior segment pathology.

**Conclusion:**

This study provided a comprehensive understanding of ophthalmic challenges faced in HA environments. Awareness of these possible manifestations, along with accurate, timely diagnosis and, if necessary, referral to a specialist, is crucial to ensure optimal ocular health for individuals stationed at high altitudes.

## Introduction

High-altitude (HA) environments present unique challenges to human physiology and health. Among the various systems affected by altitude-related exposure, the eyes are no exception. Ocular manifestations at high altitudes have been a growing concern as more individuals, particularly young adults, live and work in these regions, most of them as part of military duty requirements. Military personnel must endure prolonged postings in challenging high-altitude terrain. In addition, as awareness of health and fitness grows, more individuals are engaging in outdoor adventure sports, often in high-altitude terrain, such as mountain climbing or snow skiing [1].

The physiological changes associated with high altitudes, such as reduced oxygen levels and increased exposure to ultraviolet radiation, create an environment that can significantly compromise ocular health. The quantity of UV light increases with altitude at a rate of 4% for each 300 m ascent [2-4]. A combination of high altitude and snow at 2,000 m results in twice the amount of UV light compared to sea level [2-4].

Understanding the nature of these ocular conditions, their clinical characteristics, management, and potential causes for delayed resolution is crucial for healthcare providers and individuals alike. Under low-pressure conditions, the partial pressure of oxygen (PO2) decreases, lowering alveolar PO2 and, consequently, arterial and tissue PO2 [2-4]. The eye tissues most affected by exposure to hypoxia are the cornea and retina [2-4]. Ocular blood flow is also affected at HA [5]. Hypoxia causes corneal thickness to increase due to oedema of the corneal stroma. This is most likely due to hypoxia-induced endothelial pump deficiency [6-8].

Other ophthalmic conditions reported at high altitude include dry eye disease, meibomian gland dysfunction, increased susceptibility to ocular infections, cranial nerve palsies, and photokeratitis (snow blindness) [4,9,10]. Posterior segment pathologies, such as high-altitude retinopathy (HAR), vitreous haemorrhage, maculopathy, solar retinopathies, and macular oedema, are well-known HA manifestations [11-13].

In this study, we examined a broader range of anterior segment ophthalmic conditions among individuals exposed to high-altitude environments. By shedding light on the anterior segment ophthalmic challenges posed by high altitudes, we aimed to contribute to the body of knowledge surrounding altitude-related health issues and provide valuable insights for healthcare providers, policymakers, and individuals living and working in these unique and demanding environments.

## Methods

This is a prospective study of low-altitude (<2,400 meters above sea level) natives exposed to HA environments who presented with ocular complaints at our healthcare facility in HA (3,500 meters above sea level) in Northern India. The data was collected over 24 months (May 2022 to April 2024). The study adhered to the tenets of the Helsinki Declaration and obtained ethical approval from the institutional review board. Patient confidentiality and data anonymity were strictly maintained during data collection and analysis.

All patients who were otherwise healthy and were freshly diagnosed with anterior segment pathologies after being exposed to HA environments were included in the study. The study selectively enrolled patients who could provide documentation confirming that their systemic and ocular examinations were unremarkable before their exposure to HA, thereby ensuring a fresh diagnosis. Local inhabitants of HA, patients having posterior segment pathologies, patients unwilling to participate, and patients having pre-existing ocular conditions before being posted to HA were excluded from the study.

All patients underwent a complete ophthalmological examination, along with serological and biochemical tests, to assess any systemic association. Once the diagnosis was made, patients underwent conventional treatment for their respective anterior segment pathologies. A complete ophthalmological examination was repeated weekly for up to three months. Then, the patients were followed up three times a month for one year to note any recurrences. “Disease resolution” was considered the disappearance of all signs and symptoms. The duration of resolution was noted. Any adverse drug reaction was also noted.

Outcome measures included age, gender, duration of posting at HA, elevation of HA area, duration of symptomatology, best corrected visual acuity (BCVA) at presentation, BCVA post resolution, diagnosis based on signs and symptoms, treatment undertaken, time duration for resolution/hospital stay, associated laboratory findings, recurrences (if any) and need for de-induction/descent. Positive laboratory tests were also noted.

### Statistical Analysis

Data was entered in a Microsoft Excel sheet. Double entry and the accuracy of entered data were rechecked. SPSS version 23.0 (SPSS, Inc., Chicago, IL, USA) was used for statistical data analysis. To assess normality, the Shapiro-Wilk test was used. Categorical variables were presented as percentages, and continuous variables as mean ± standard deviation (SD). Student’s t-test was used for pre- and post-BCVA comparisons. Fisher’s exact test was used to evaluate recurrences/adverse events. The confidence interval was kept at 95%. A p-value of < 0.05 was considered statistically significant.

## Results

A total of 2,043 patients visited the ophthalmology outpatient department during the study period, presenting with ocular complaints. Out of these, 130 individuals met the inclusion criteria. The mean age of the study population was 25.67 ± 5.14 years. All were males, owing to their occupational requirements (security personnel). The mean elevation was 3,560 meters above sea level.

**Fig. 1:**
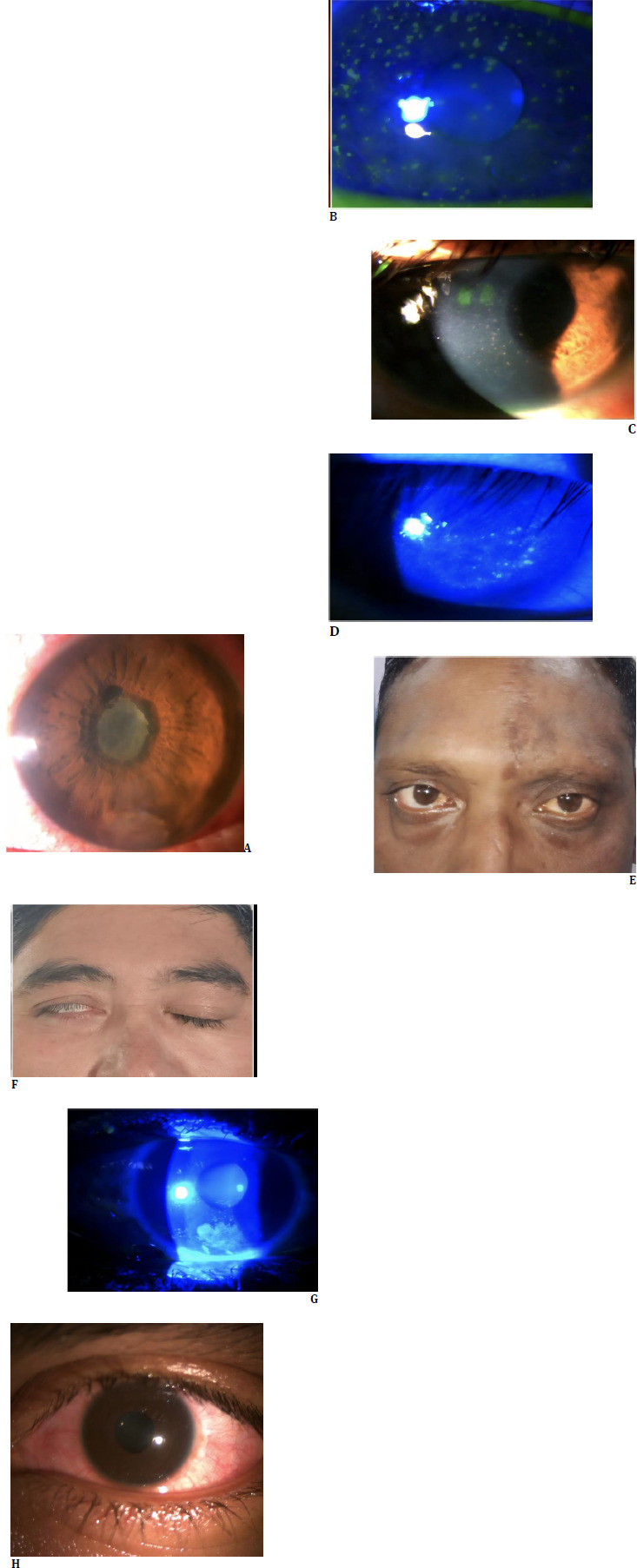
Anterior segment manifestations in native lowlanders exposed to high altitude (**A**) Acute anterior uveitis associated with severe anterior chamber reaction and pupillary membrane formation; (**B**) Herpes simplex virus (HSV) keratitis; (**C**) HSV keratitis with iridocyclitis; (**D**) Photokeratitis; (**E**) Herpes Zoster Ophthalmicus left side; (**F**) Facial nerve palsy right side; (**G**) Exposure keratopathy associated with facial nerve palsy; (**H**) Non-resolving bilateral chronic conjunctivitis

The anterior segment manifestations, that were encountered, included acute anterior uveitis (AAU) (n=12), Herpes Simplex Virus (HSV) Keratitis (n=30), HSV Keratitis with Iridocyclitis (n=4), Photokeratitis (n=30), Herpes Zoster Ophthalmicus (HZO) (n=10), facial nerve palsy with or without exposure keratopathy (n=24), non-resolving/chronic inflammatory conjunctivitis (n=20) (**Fig. 1**). The mean duration of stay at HA ranged from 10 weeks to 21 weeks.

**Table 1** lists demographic and high-altitude exposure characteristics of all anterior segment ophthalmic conditions and cranial nerve palsies. **Table 2** presents the visual acuity and symptom profile of anterior segment manifestations, while **Table 3** lists clinical outcome parameters. HLA-B27 was positive in 83.33% of AAU cases. Raised total leucocyte count (TLC), specifically lymphocyte count, was found in HSV keratitis (5247 ± 364.28/micro-liter), HSV keratitis with iridocyclitis (5054 ± 386.18/micro-liter), and HZO (5115 ± 387.07/micro-liter) cases. Most cases responded well to treatment. However, 50% AAU cases (n=6), 25% of HSV keratitis cases (n=6), 20% of HZO cases (n=2), and 33.33% of facial nerve palsy cases (n=8) suffered recurrence within a period of one year, and therefore, had to be de-inducted to a lower altitude. De-induction of these patients led to immediate resolution of their condition. Conditions, such as HZO and chronic inflammatory conjunctivitis, had little to no effect on presenting visual acuity. However, other ophthalmic manifestations led to marked blurring of vision at presentation. The conservative treatment modalities used produced a statistically significant improvement in BCVA in these conditions (p<0.05). No adverse event to any of the drugs was found.

**Table 1:** Demographic and High-Altitude Exposure Characteristics of Anterior Segment Manifestations

S. No.	Diagnosis	No. of cases	Mean duration of residing at HA (weeks)	Mean elevation of HA area (meters)
1	**AAU**	12	12.3	3560
2	**HSV keratitis**	30	11.5	3500
3	**HSV keratitis with AAU**	4	10.6	3620
4	**Photokeratitis**	30	12.2	3620
5	**HZO**	10	15.5	3500
6	**7^th^CN Palsy**	24	12.6	3560
7	**Chronic inflammatory conjunctivitis**	20	20.6	3560

* statistically significant; AAU = Acute anterior uveitis, HSV = Herpes simplex virus, HZO = Herpes zoster ophthalmicus, CN = Cranial Nerve, No. = Number

**Table 2:** Visual Acuity and Symptom Profile of Anterior Segment Manifestations

S. No.	Diagnosis	Mean duration of symptoms ± SD (days)	Mean BCVA at presentation (logMAR)	Mean BCVA after treatment (logMAR)	P-value
1	**AAU**	7.03 ± 0.02	0.72 ± 0.02	0.11 ± 0.01	<0.05*
2	**HSV keratitis**	6.91 ± 0.10	0.51 ± 0.01	0.09 ± 0.01	<0.05*
3	**HSV keratitis with AAU**	12.01 ± 1.10	0.63 ± 0.10	0.10 ± 0.04	<0.05*
4	**Photokeratitis**	2.01 ± 0.01	0.34 ± 0.01	0.01 ± 0.01	<0.05*
5	**HZO**	4.20 ± 0.51	0.30 ± 0.02	0.22 ± 0.01	>0.05
6	**7^th^CN Palsy**	6.01 ± 1.1	0.33 ± 0.01	0.02 ± 0.01	<0.05*
7	**Chronic inflammatory conjunctivitis**	27.12 ± 1.2	0.21 ± 0.01	0.19 ± 0.03	>0.05

* statistically significant; AAU = Acute anterior uveitis, HSV = Herpes simplex virus, HZO = Herpes zoster ophthalmicus, CN = Cranial Nerve, No. = Number

**Table 3:** Clinical Outcome Parameters of Anterior Segment Manifestations at High Altitude

S. No.	Diagnosis	Mean duration of resolution ± SD (days)	Associated laboratory findings	Recurrences (number of cases)	Need for de-induction (% patients)
1	**AAU**	30.50 ± 7.80	HLA B-27+ in 83.33 % cases	3	50%
2	**HSV keratitis**	25 ± 5.16	RAISED TLC	3	25%
3	**HSV keratitis with AAU**	35.02 ± 6.12	RAISED TLC	-	-
4	**Photokeratitis**	009.70 ± 0.12	-	-	-
5	**HZO**	40.02 ± 3.50	RAISED TLC	1	20%
6	**7^th^CN Palsy**	45 ± 6.40	-	4	33.33%
7	**Chronic inflammatory conjunctivitis**	30.65 ± 8.09	-	-	-

* statistically significant; AAU = Acute anterior uveitis, HSV = Herpes simplex virus, HZO = Herpes zoster ophthalmicus, CN = Cranial Nerve, No. = Number

## Discussion

The results highlighted the spectrum of anterior segment conditions, including HSV keratitis and uveitis. The prolonged resolution of conditions such as chronic inflammatory conjunctivitis underscores the importance of early diagnosis and prompt management. We aimed to highlight the varied ophthalmic manifestations of the anterior segment of the eye that are aggravated or manifest in HA areas. To the best of our knowledge, no prior study has examined anterior segment manifestations and cranial nerve palsies in HA areas. There has been sufficient research on posterior segment manifestations [11-13].

We observed an increase in cases of herpetic eye disease. This may be due to reduced immunity in HA areas, which is associated with cold weather and increases the risk of viral infections [14,15]. Although HA areas are known to have higher infection rates, no previous case reports or articles have documented herpetic eye diseases in these areas. HSV keratitis, if untreated for long, may lead to permanent sequelae, such as corneal opacities, which are potentially sight-threatening. Therefore, early treatment is warranted.

The mean duration of symptoms across these conditions ranged from 2 to 27 days. The prolonged duration of symptoms in cases of non-resolving conjunctivitis may be due to misdiagnosis by local physicians. Changes in the normal flora of the conjunctiva at high-altitude areas, in association with changes in ocular surface parameters, may be the cause of this condition [14,16]. Hence, broad-spectrum antibiotics (Polymyxin B + Chloramphenicol) in the form of eye ointment were prescribed, which led to complete resolution within approximately one month. We also observed a whopping 24 cases of facial nerve palsy. Certain previous case reports have also described cranial nerve palsies at HA areas, including pupil-sparing third nerve palsy and facial nerve palsy [10,17,18]. Reasons for facial palsy that are specific to high altitude include hypoxia, cold weather, vitamin D deficiency, and high-altitude cerebral oedema [10,17,18]. However, unlike the above case reports, immediate de-induction was not required in our cases. De-induction was required only in cases that recurred within one year (33.33%). In our study, many newly diagnosed AAU cases (n=12) were also observed (considering the short period of observation), the majority of which were associated with HLA-B27 (83.33%). No such cases at HA have been reported. It can be hypothesized that HA and cold environments may aggravate inflammatory eye conditions [2-4]. One reason for this finding may be a dysfunctional immune system in HA areas [4,14]. Low oxygen partial pressure, exposure to ultraviolet (UV) light, and very low temperatures can affect the immune system and increase susceptibility to various infectious and autoimmune diseases [4,14,15].

Photokeratitis was observed in 30 individuals. It is important to differentiate between herpetic keratitis and photokeratitis. Photokeratitis usually resolves within 24-48 hours with rest, protective goggles, and lubricants [2,4]. Increased UV exposure, associated with increased light reflectivity in snow, leads to this condition [1-4]. However, this condition is not sight-threatening and almost always resolves with conventional treatment.

Overall, the findings from this study provided valuable insights into the ocular health challenges faced by individuals exposed to high altitudes and emphasized the importance of tailored healthcare strategies for this unique population. The results of this study shed light on the diverse spectrum of these conditions, their demographics, clinical characteristics, and the potential factors contributing to delayed resolution. Nevertheless, the study had its limitations. The study was conducted in a specific HA healthcare facility, which might have limited the generalizability of findings to other HA regions.

However, the findings of this study had several implications for healthcare delivery in HA regions. Firstly, there was a clear need for well-equipped healthcare facilities staffed with ophthalmologists capable of managing a wide range of ocular conditions. Secondly, preventive measures, such as protective eyewear and eye health education, should be implemented to reduce the incidence of ocular conditions associated with HA exposure. Additionally, telemedicine and remote consultation options might help bridge the gap in healthcare access, especially in remote HA locations. Further research and collaborative efforts are warranted to enhance healthcare delivery and address the evolving demands of HA environments.

## Conclusion

Ophthalmic manifestations in high-altitude environments are a complex and multifaceted challenge. This study provided valuable insights into the nature of these conditions, their demographics, and the factors influencing their resolution. Timely diagnosis, specialist referral, and preventive strategies are key to preserving ocular health in individuals exposed to high altitudes.
